# Incidence, pattern and mechanisms of injuries and fractures in children under two years of age

**DOI:** 10.1186/s12891-021-04420-4

**Published:** 2021-06-18

**Authors:** Karen Rosendahl, Ramona Myklebust, Kjersti Foros Ulriksen, A. Nøttveit, Pernille Eide, Åsmund Djuve, Christina Brudvik

**Affiliations:** 1 Faculty of Health Sciences, Department of Clinical Medicine, UiT the Arctic University of Norway, Tromsø, Norway; 2 Section of Paediatric Radiology, University Hospital North Norway, Pb 100, 9038 Tromsø, Norway; 3 Norheimsund Medical Center, Norheimsund, Norway; 4 Triangel Medical Center, Stjørdal, Norway; 5 Bergen Accident and Emergency Department, Bergen, Norway; 6 Knarvik Medical Center, Knarvik, Norway; 7 Harstad Hospital, Harstad, Norway; 8 Department of Clinical Medicine, K1, University of Bergen, Bergen, Norway

## Abstract

**Background:**

Fractures in children under 2 years of age are rare, and little has been published on their mechanisms. We aimed at examining the incidence, mechanisms, pattern and fracture characteristics in a large, population-based cohort of otherwise healthy children.

**Methods:**

This retrospective, cross-sectional study includes all children aged 0–2 years, attending the Accident and Emergency department in Bergen between 2010 and 2015, due to an injury warranting radiography. Clinical data was categorized from the medical notes, and all radiographs were reviewed by an experienced paediatric radiologist.

**Results:**

In total 408 children (212 male), 3–23 months of age (mean 17.7 months), were included. 149 (77 male) children had a total of 162 fractures, yielding an annual incidence of 5.4 per 1000, varying from 0.7 per 1000 for those under 12 months of age, increasing tenfold to 7.3 per 1000 for children aged 12–24 months of age. More than half of the fractures (53.1%) were seen in children aged 18–23 months, while none was found in those under 7 months of age. The youngest age group had mostly femur and tibia fractures, the oldest mostly forearm fractures (*n* = 55, 33.9%), followed by tibia fractures (21.6%) and fractures to the clavicle (14.8%). The reported mechanisms for the 162 fractures were fall from a chair/bed/table (41.4%), fall from own height (18.5%) or crush injury (15.4%). In 8 of 162 (4.9%) fractures, the history was clearly inconsistent and suspicious of non-accidental injury (NAI).

**Conclusion:**

Injuries and fractures in young children in general, and non-ambulant children in particular, are rare and should be thoroughly assessed for NAI. *Level of evidence:* Retrospective, population based cross-sectional study. Level 3.

## Introduction

The annual incidence rates of fractures in children under 16 years varies from 3.6 per 1000 to 50 per 1000 according to age, gender, social and environmental factors, and typically peaks at 11–12 years for girls and 13–14 years for boys [1–5]. The male-to-female incidence ratio is 1.5 [5]. The distal forearm is the most affected site, and often caused by a fall [2, 5]. In children under 2 years of age the numbers are low, particularly in non-ambulatory children, with a predilection for the clavicle and skull in those under 8 months of age [1]. In children between 9 and 24 months of age, forearm, tibia and fibula fractures predominate [1].

We have previously shown, in a population-based cohort of 408 children under 2 years of age, that 149 (77 boys) children had a total of 162 fractures, yielding a fracture incidence of 5.9 per 1000 and an incidence of children with fractures of 5.4 per 1000 [6]. Fractures to the forearm were the most common, accounting for one third of the fractures, followed by tibia and clavicle. One epiphyseal separation in the left first metatarsal, and one metaphyseal lesion in the proximal left humerus without a history of trauma were also identified. We here report details on injury and fracture incidence, pattern and mechanisms from the same cohort [6].

## Methods

This is a retrospective, cross-sectional study. All children under the age of two, attending Bergen Accident and Emergency Department (BLV) due to an injury during May 20th 2010 to April 1st 2015, were eligible for the study. Included were those having radiographs taken. BLV is the only A&E department in Bergen and its surrounding municipalities examining children with suspected fractures. Excluded were children with birth related fractures and children with major trauma, admitted directly to the emergency unit at the University Hospital. The patients were identified through searches in the PACS system (Picture and Archiving Communication System) (Impax 6, Agfa-Gevaert, Belgium) at the radiology department, Haukeland University Hospital. Data on demographics, time from injury to examination, month of injury and injury mechanisms were collected from the medical notes at BLV, and registered in an anonymous form by five of the co-authors under guidance of a senior A&E physician and GP (CB). All radiographs were taken on an Intuition DR system (Arcoma AS, Sweden) and, in a later session using a high-resolution PACS screen, reviewed by five of the co-authors and a senior paediatric radiologist (KR). The following features were registered: anatomic region (bone), type (complete (simple/wedge, complex), incomplete (bowing, greenstick/buckle), other (classic metaphyseal lesion (CML), avulsion, fissure), healing signs (periosteal new bone formation / callus (no/yes)), bone structure (osteopenia) no/yes) and metaphyseal appearances (published elsewhere [6]). For long bone fractures we also registered which segment was involved (proximal, shaft, distal) according to an adjusted version of the Müller classification [7]. A fissure was defined as a lytic line within the bone with no visible involvement of the cortex, evidenced by a periosteal reaction at follow-up after around 10–14 days. Paired fractures to the tibia/fibula or radius/ulna were registered as two fractures. The presence of osteopenia was assessed subjectively, based on the thickness of the cortex, and on the appearances of the trabeculae [8–10]. Ethical approval of the study, including the need for informed consent, was waived by the Regional Ethical Committee (REK-N, no. 2012/172), University of Bergen, the Medical Faculty, post-box. 7804, 5020 Bergen, Norway. All methods were carried out in accordance with relevant guidelines and regulations.

Descriptive statistics was used in the analysis of demographic data. In order to access incidence rates, the annual number of children under 2 years of age residing in Bergen during the study period was retrieved from Statistics Norway (https://www.ssb.no/population). Study size was based on the expected fracture rates, to secure appropriate numbers across types. Differences in the number and types of injuries, and the number, sites and types of fractures between males and females and according to place and time of the year were examined using Pearson Chi-Square test (2-sided) or Fisher’s exact test as appropriate. A *p*-value < 0.05 was considered statistically significant. Statistical analyses were performed using IBM SPSS (Statistical Package for the Social Sciences) Statistics, version 26.

## Results

**Fig. 1:**
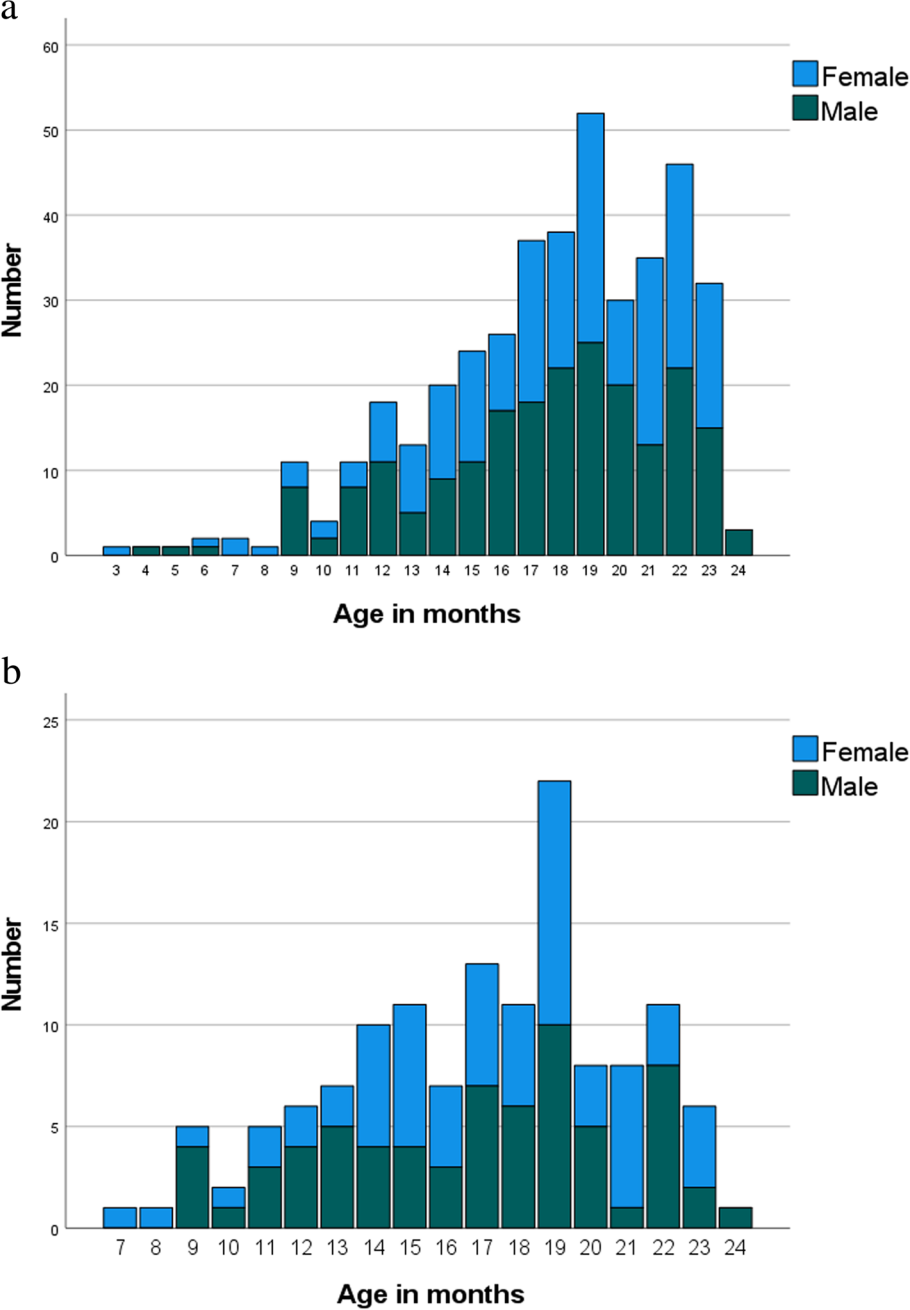
**a** Number of otherwise healthy children aged 0–2 years sustaining an injury warranting an x-ray (*n* = 408) during the period 2010–2015, and **b** children with fractures (*n* = 149) from the same population, by age and gender (red = female, blue = male)

A total of 408 children (212 boys) (mean age 17.7 months, range 3–23 months) were included (Fig. 1a). One hundred sixty-two fractures were identified in 149 (77 male) of the 408 children (36.5%), yielding an annual incidence of 5.4 per 1000, varying from 0.7 per 1000 for those under 12 months of age to 7.3 per 1000 for those aged 12–24 months (Fig. 1b). There were no differences in fracture incidence according to gender (*p* = 0.486). Except for five children with asthma and two with epilepsy, none of the children had chronic disease. Two of the 408 children had sustained a previous fracture. The bone structure was considered normal radiographically in 403 out of the 408 (98.8%) children, while five showed focal bone loss.

### Injury mechanisms

109 (26.7%) of the 408 injuries were caused by a fall from furniture (chair/table/bed), while 103 (25.3%) were caused by a crush injury and 62 (15.2%) by a fall from the child’s own height (Table 1). In 60 of 408 injuries (14.7%), the mechanism was unknown, ranging from 2 out of 3 (67.7%) of those aged 0–5 months, 2/31 (6.5%) of those aged 6–11 months, 17/138 (12.3%) of those aged 12–17 months and 39/236 (16.5%) of those aged 18–23 months (*p* = 0.000, Chi-squared test).

**Table 1:** Mechanism and localization of injury in 408 children aged 0–2 years with suspected and verified fractures. One hundred forty-nine children had 162 verified fractures

Fracture localization	Fall from chair/ bed / table	Fall from own height	Crush -injury	Trampoline	Stretch / pull	Dropped by parent	Direct blow	Twisting	Unknown	Other mechanisms	Total (%)
Humerus	7	2	0	1	0	0	1	0	1	0	13^a^ (8.0)
Radius	24	9	0	0	0	0	0	0	4	0	37 (22.8)
Ulna^b^	10	6	0	0	0	0	0	0	2	0	18 (11.1)
Metacarpal	0	0	2	0	0	0	0	0	0	0	2 (1.2)
Phalanx hand	0	0	17	0	0	0	0	0	2	0	19 (11.7)
Femur	2	1	0	0	0	2	0	0	0	0	5 (3.1)
Tibia	5	7	2	2	3	2	0	2	5	0	28 (17.3)
Fibula	2	0	0	0	1	1	0	0	3	0	7 (4.3)
Metatarsal	2	1	1	1	0	0	0	0	0	0	6^b^ (3.7)
Phalanx foot	0	0	2	0	0	0	0	0	1	0	3^c^ (1.9)
Clavicle	14	4	1	0	0	0	1	0	4	0	5 (3.1)
Total number of fractures	67	30	25	4	4	4	2	2	22	0	162 (100.0)
No fracture	42	32	78	7	21	1	7	1	38	19	246
Total number of children	109 (26.7)	62 (15.2)	103 (25.3)	9 (2.2)	29 (7.1)	5 (1.2)	9 (2.2)	3 (0.7)	60 (14.7)	19 (4.7)	408

^a^ Including a metaphyseal lesion (CML)

^b^ None of the ulna fractures were isolated fractures, but combined antebrachii fractures

^c^ Including an epiphyseal separation

The reported mechanisms for the 162 fractures were fall from chair/bed/table (*n* = 67, 41.4%), fall from own height (*n* = 30, 18.5%) and crush injury (*n* = 25, 15.4%). 17 (68%) out of 25 crush injuries involved the distal phalanges of the hand. In 13.6% of the fractures, the mechanism was unknown (Table 1).

The reported injury mechanisms differed significantly between the non-fracture and the fracture group (*p* = 0.000). Significantly more children with a fracture had been injured by a fall from furniture (67/162, 41.4%) or from the child’s own height (30/162, 18.5%) than children without a fracture (42/246, 17.1%, and 32/246, 13.0%, respectively). However, children with crush injuries less often had a fracture, despite the clinical suspicion that had warranted an x-ray (Table 1).

### Environment and season of injury

Of the 408 injuries, 133 (32.6%) were sustained at home while 18.9% took place in the kindergarten. In 42.4% of the injuries, the caregiver(s) did not know, or were uncertain where the accident had happened. No differences in place of the injury or time of the year was seen according to gender (*p*-values 0.796 and 0.759, respectively).

### Time from injury to medical attendance

A total of 212 / 408 children (52%) were brought to the A&E within 6 h of the injury, 311 (76.3%) within 24 h, and 331 (81.1%) within 48 h. Seventeen children (4.2%) attended BLV after more than 3 days (of whom 5 had fractures). In 49 cases (12% of all injuries), the interval between injury and visit was unknown, of whom 18 had fractures. There were no differences according to gender (*p* = 0.186).

Signs of fracture healing were evident in five, otherwise healthy children (mean age 14.6 months, range 9–18 months) for whom the interval between injury and visit was unknown in two; a 9 months old boy with a distal femur fracture and a 17 months old girl with fractures to the distal tibia and fibula. The remaining three had allegedly sustained their fractures within the past 72 h; an 18-months-old with a clavicle fracture, a 14-months-old with a greenstick fracture to the tibia and a 14-months-old with a greenstick fractures to the distal radius/ulna. The reported fracture mechanism was fall from low heights in all five, except for a 9-months-old with a femur fracture, in whom the parents offered no explanation.

*An inconsistent fracture history,* i.e. a mismatch between the fracture mechanism offered by the caretaker and the radiographic findings, was considered in 29 (19.5%) children having sustained a fracture, of which 8 (5.4%) (5 males) were clearly inconsistent (7/8 were aged 6–17 months).

### Fracture symptoms and mechanisms

The most frequent symptoms amongst the 149 children with fracture were an avoidance response in 52 (34.9%), pain in 31 (20.8%) and crying/uneasiness in 20 (13.4%). In 28 children (18.8%), no symptoms were recorded (16 crush injuries, 8 falls from low height, 4 unknown) ranging from 25.0% in those aged 6–11 months to 18.1% in those aged 18–23 months (*p* = 0.050, Fisher’s exact).

### Fracture location and type

Of the 162 fractures, 55 (33.9%) involved the forearm, followed by tibia fractures (17.3%), fractures to the clavicle (14.8%), to the hand (12.9%) and to the foot (5.6%) (Table 2). 42.0% of the fractures were complete (simple, wedge, complex), while 32.1% were greenstick or buckle fractures, 12.1% were avulsions and 6.8% were fissures. There was one epiphyseal separation and one CML. No gender differences were seen according to fracture site (*p* = 0.138) or fracture type (*p* = 0.281) (both Fisher’s exact). Most of the long bone fractures were located distally; 84% of the humerus-, 64.9% of the radius, 83.3% of the ulna, 82.1% of the tibia and all the fractures to the femur and to the fibula (Table 2).

**Table 2:** Localization by gender for 162 fractures in 149/408 children 0–2 years of age, admitted to the A&E department due to an injury

Localization	Female	Male	Total (%)
*Upper extremity*			
Humerus	9	3	13^a^ (8.0)
-Proximal	0	1	1
-Shaft	0	1	1
-Distal	9	2	11
Radius	19	18	37 (22.8)
-Proximal	0	1	1
-Shaft	6	7	13
-Distal	13	11	24
Ulna	12	6	18 (11.1)
-Proximal	0	0	0
-Shaft	2	1	3
-Distal	10	5	15
Metacarpal	2	0	2 (1.2)
Phalanx hand	8	11	19 (11.7)
*Lower extremity*			
Femur	3	2	5 (3.1)
-Proximal	0	0	0
-Shaft	0	0	0
-Distal	3	2	5
Tibia	11	17	28 (17.3)
-Proximal	3	2	5
-Shaft	0	0	0
-Distal	8	15	23
Fibula	5	2	7 (4.3)
-Proximal	0	0	0
-Shaft	0	0	0
-Distal	5	2	7
Metatarsal	1	4	60^b^ (3.7)
Phalanx foot	1	2	3 (1.9)
*Other*			
Clavicle	11	13	24 (14.8)
Total number of fractures (%)	82 (50.6)	78 (49.4)	162 (100.0)

^a^ Including one metaphyseal lesion (CML)

^b^ Including an epiphyseal separation

Of the 162 fractures, 86 (53.1%) were seen in children aged 18–23 months, while none were seen in children under 7 months of age (Fig. 1b) (Table 3). In children aged 6–12 months, femur and tibia fractures were most common, in children aged 12–18 months tibia and forearm fractures were most common, and in the oldest age group aged 18–24 months, forearm fractures predominated by large (Table 3).

**Table 3:** Localization by age group for 162 fractures in 149/408 children 0–2 years of age, seen at the A&E department due to an injury

Localization	0–6 months	6–12 months	12–18 months	18–24 months	Total (%)
*Upper extremity*					
Humerus	0	2^a^	3	8	13^a^ (8.0)
Radius	0	1	15	21	37 (22.8)
Ulna^b^	0	0	7	11	18 (11.1)
Metacarpal	0	0	2	0	2 (1.2)
Phalanx hand	0	2	7	10	19 (11.7)
*Lower extremity*					
Femur	0	4	1	0	5 (3.1)
Tibia	0	3	14	11	28 (17.3)
Fibula	0	0	4	3	7 (4.3)
Metatarsal	0	1	0	5	6^c^ (3.7)
Phalanx foot	0	1	1	1	3 (1.9)
*Other*					
Clavicle	0	1	7	16	24 (14.8)
Total number of fractures (%)	0	13 (8.0)	63 (38.9)	86 (53.1)	162 (100.0)

^a^ Including one metaphyseal lesion (CML)

^b^ None of the ulna fractures were isolated, but antebrachii fractures

^c^ Including an epiphyseal separation

#### Femur fractures

A total of five femur fractures were identified. All five fractures were located distally; four were seen in infants aged 7–9 months (3 girls) whereas the fifth was found in a 14 months old girl. Two were stable greenstick-fractures and three were complete fractures. One of the children was further assessed in hospital because of suspicion of non-accidental injury. An additional older fracture to the clavicle was found in hospital. All five fractures were allegedly caused by fall from either parents’ arms, stairs or changing tables.

## Discussion

We have shown that fractures are rare in otherwise healthy children under the age of 12 months, with a 10-fold increase in those between 12 and 24 months. Moreover, that fractures to the femur and tibia predominate in the youngest as opposed to tibia and forearm fractures in the oldest age group, with an increase in forearm fractures with increasing age. While falls from a low height was the most commonly reported mechanism amongst those sustaining a fracture, crush-injuries predominated in children without a fracture. In 13.6% of the fractures, the mechanism was unknown.

The annual fracture incidence of 5.4 per 1000 found in the current study did not differ according to gender, as opposed to a male predominance seen in older children [2, 11, 12]. Except for a study by Clarke et al., reporting a similar fracture incidence of 5.3 per 1000 children under the age of two [1], the number of epidemiological studies addressing fracture rates, types and mechanisms in otherwise healthy infants aged 0–2 is sparse. This contrasts the substantial body of studies on children and adolescents up to 19 years of age [4, 5, 11, 13–17], of which a few report on figures for those under two, specifically [11, 15]. In his classical study from 1983, Landin found that fractures to the clavicle predominated in children under the age of two, with incidences of 1.8 and 2.2 per 1000 for boys and girls, respectively, followed by fractures to the skull and tibia [11]. In a more recent study from 2007, Rennie et al. reported a fracture incidence of 3.6 per 1000 infants under the age of one. Again, fractures to the clavicle predominated [15]. Similar findings have been reported by others, however, with no incidences given [18, 19].

We found that most of the long bone fractures were located distally, a finding also reported by others [1]. Further, fissures were most often seen in the distal tibia in children aged 12–24 months, consistent with Toddler’s fractures in ambulatory children. Occasionally, these fractures are very subtle and may be missed radiographically. In cases with a mis-match between symptoms and findings, a follow-up radiograph after 2 weeks can help to establish the diagnosis.

As opposed to others [1, 11, 15], we did not see any fractures in infants under 7 months of age, however, infants sustaining head- or high energy injuries were not included in our series as these children are routinely admitted to hospital. Thus, it is reasonable to believe that the occurrence of these fractures types is relatively similar in our population.

Of note is that nearly all children included in our study were otherwise healthy, with a normal bone structure judged radiographically. Still, most of both fracture-suspected injuries and fracture injuries in our study were due to low energy trauma, in this particular setting caused by falls from chairs, tables or beds, or falls from the child’s own height, as opposed to traumas caused by car accidents or falls from heights. However, the distribution of fractures, with femur fractures predominating in infants younger than 1 year of age is intriguing. Our estimated incidence rate of 0.36 per 1000 was significantly higher than that reported in a recent study from England [20]. This study, including 1852 closed, isolated femoral shaft fractures in children aged 0–15 years, reported a mean annual incidence rate of 0.06 (95% CIs 0.02–0.10) per 1000 population for children aged < 1 year, rising to 0.12 (0.08–0,16) for those aged 1–2 years. The age of peak incidence was 2 years for both boys and girls, decreasing with increasing age. Falls less than two metres was the most common injury mechanism across all age categories, but this was most pronounced in the 18 months to 3 years age category. Unfortunately, the TARN (Trauma Audit & Research Network / NHS) database does not include the exact height fallen, nor was there any information about the child’s mobility. The authors state that most falls in toddlers represent a low energy impact which can result in spiral femoral shaft fractures. Their study found non-accidental injury (NAI) to be a suspected cause of femoral fractures in 3.8% of children. In contrast, one of five femur fractures in our cohort was suspect of NAI. The child, a 7 months old girl with an oblique/spiral fracture to the distal femur, was allegedly dropped onto the floor by a parent. She was admitted to hospital, where a skeletal survey showed an additional old fracture to the left clavicle. The remainder four femur fractures were seen in three non-mobile children aged 7–9 months, and in one 14-months-old, caused by falls from low heights/child’s own height or dropped by a parent. According to existing literature, a child sustaining a femur fracture has approximately a 1 in 3 chance of having being abused, and femur fractures resulting from abuse are more commonly seen in children who are not yet walking [21, 22]. This knowledge is mirrored in our national guidelines, having a low threshold for performing a skeletal survey in infants presenting with a femur fracture with no plausible explanation being offered [23].

Around half of the fractures were seen in children aged 18–24 months, with forearm and tibia/fibula fractures accounting for around 60%; findings that are in line with those reported by Clarke [1]. The mechanism of these fractures was primarily fall from furniture or own height. In nearly 15% of the fractures, no injury mechanism was offered, a figure that should be read with caution due to the retrospective nature of our study. Of note is, however, that an inconsistent fracture history was considered in almost 20% of the children as compared to 15% in Clarke’s study. Some of these children and their families were referred to the child protection service (CPS) for further assessments, according to national guidelines.

Similarly, an unexplained delay in presenting to an emergency department following an injury can be indicative of abuse or maltreatment [24, 25]. In our series, more than 50% of the 408 injured children were brought to the BLV within 6 h of the injury, rising to 76% within 24 h, as compared to 27% and around 50%, respectively, in the study by Clarke [1]. Seventeen children attended BLV after more than 3 days, of whom 5 had fractures, and in 49 cases, the interval between injury and visit was unknown, of whom 18 had fractures. It is unclear how many of these children were referred to CPS, underscoring the importance of accurate and detailed medical notes in infants presenting with a fracture. In a study by Banaszkiewicz et al., the authors conclude that in 28%, abuse had been initially underestimated as a cause of injury [26]. In order to systematically address possible NAI, new prospective studies with generalized forms and standardized follow up routines, could have the potential to identify, address and help young children and their families at an early stage after injury. However, the need for declarations of consent, is a limitation to this type of study.

We acknowledge several limitations to our study - firstly, there is its retrospective nature prone to missing or incomplete data. Secondly, we did not validate the classification of fractures prior to analysing the radiographs, however, this was not our intention with this study. Thirdly, we did not include new-borns sustaining birth injuries or infants sustaining high energy injuries, as these were admitted directly to the emergency unit at the University hospital. The strengths of this study include the detailed review of all clinical data, the detailed consensus review of all the radiographs, high-resolution images and the population-based approach.

## Conclusion

The incidence of fractures in otherwise healthy children was low, with no fractures seen in those under 7 months of age. Fracture to the distal femur predominated between 6 and 11 months-of-age as opposed to tibia - and later forearm fractures in children between 12 and 23 months-of-age. Around 60% of fractures were caused by falls from low heights, but the fracture histories were clearly inconsistent and suspicious of NAI in 5%, underlining the need for accurate and detailed medical notes in young children with suspected fractures.

## Data Availability

The datasets used and/or analysed during the current study are available from the corresponding author on reasonable request.
